# Circular RNAs: Promising Biomarkers for Age-related Diseases

**DOI:** 10.14336/AD.2020.0309

**Published:** 2020-12-01

**Authors:** Yan-hong Pan, Wei-peng Wu, Xing-dong Xiong

**Affiliations:** ^1^Guangdong Provincial Key Laboratory of Medical Molecular Diagnostics, Institute of Aging Research, Guangdong Medical University, Dongguan 523808, China; ^2^Institute of Biochemistry & Molecular Biology, Guangdong Medical University, Zhanjiang 524023, China.

**Keywords:** circRNAs, biomarker, aging, age-related diseases

## Abstract

Aging is a complex biological process closely linked with the occurrence and development of age-related diseases. Despite recent advances in lifestyle management and drug therapy, the late diagnosis of these diseases causes severe complications, usually resulting in death and consequently impacting social economies. Therefore, the identification of reliable biomarkers and the creation of effective treatment alternatives for age-related diseases are needed. Circular RNAs (circRNAs) are a novel class of RNA molecules that form covalently closed loops capable of regulating gene expression at multiple levels. Several studies have reported the emerging functional roles of circRNAs in various conditions, providing new perspectives regarding cellular physiology and disease pathology. Notably, accumulating evidence demonstrates the involvement of circRNAs in the regulation of age-related pathologies, including cardio-cerebrovascular disease, neurodegenerative disease, cancer, diabetes, rheumatoid arthritis, and osteoporosis. Therefore, the association of circRNAs with these age-related pathologies highlights their potential as diagnostic biomarkers and therapeutic targets for better disease management. Here, we review the biogenesis and function of circRNAs, with a special focus on their regulatory roles in aging-related pathologies, as well as discuss their potential as biological biomarkers and therapeutic targets for these diseases.

## 1. Introduction

Aging is an ineluctable biological process primarily regulated by several evolutionary conserved mechanisms [1, 2]. It is characterized by a progressive loss of physiological integrity caused by the cellular and molecular damage accumulation, resulting in impaired bodily functions and increased susceptibility to diseases [3, 4]. Despite accumulating evidence demonstrating that aging is a major risk factor for human diseases, the molecular mechanisms underlying this process and its link to these diseases are unknown [2, 5]. Therefore, it is important to identify the molecules that play key roles in the aging process, as well as their function in the development of age-related diseases.

Circular RNAs (circRNAs), a novel type of universal and diverse endogenous transcripts that has been a recent focus in the transcriptomics field, were first identified in viroid in 1976 [6] and first observed through an electron microscope in the cytoplasm of eukaryotic cell in 1979 [7]. CircRNAs form covalently closed loop structures with neither 5′-3′ polarities nor polyadenylated tails, and therefore, are more stable than linear RNAs and insusceptible to degradation by RNA exonuclease or RNase R [8-10]. Subsequent reports revealed that circRNAs can act as miRNA sponges, transcriptional regulators, binding partners of proteins, or even translated into functional proteins [11-15]. Furthermore, circRNAs are abundant, relatively stable, specifically expressed in tissues, and evolutionary conserved among species, affording them the potential to be biomarkers for human diseases [8, 16, 17].

Recent studies have identified several circRNAs as regulators of various pathways that are involved in aging and cellular senescence [18-21]. In particular, dysregulated circRNAs were implicated in the pathophysiology of age-related diseases, including cardio-cerebrovascular disease, neurodegenerative disease, cancer, diabetes, rheumatoid arthritis, and osteoporosis [22-24]. Here, we review the biogenesis and function of circRNAs and their potential as biomarkers of age-related diseases.

**Figure 1.**
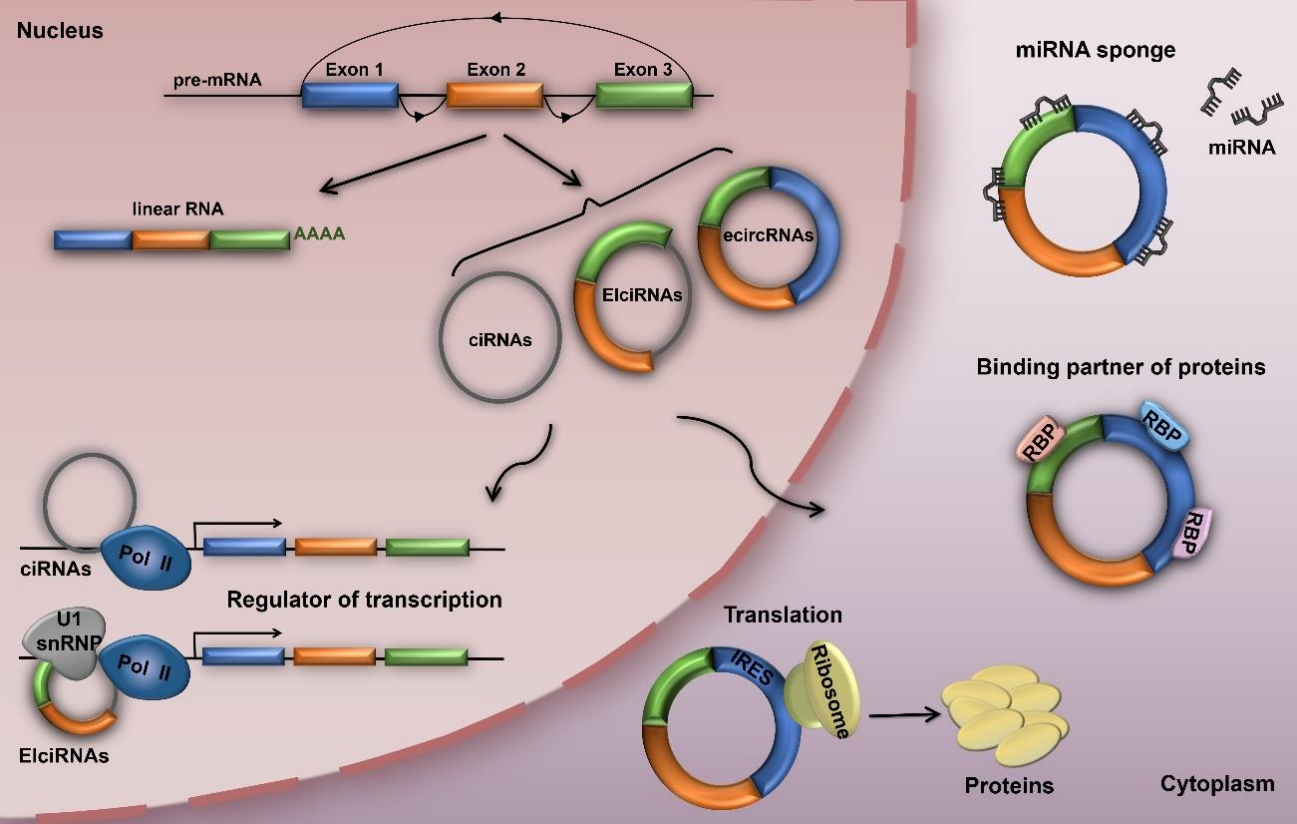
The biogenesis and function of circular RNAs (circRNAs). CircRNAs can be classified into three types - exonic circRNAs (ecircRNAs), retained-intron circRNAs or EIciRNAs and intronic circRNAs (ciRNAs). Nuclear circRNAs can regulate parental gene transcription, while cytoplasmic circRNAs can act as miRNA sponges, transcriptional regulators, binding partners of proteins, or even translated into functional proteins.

## 2. Biogenesis and function of circRNAs

CircRNAs are derived from precursor mRNAs (pre-mRNAs), which are ubiquitous in eukaryotic cells, transcribed by RNA polymerase II (RNA Pol II) [13, 25-27]. Recent studies have revealed that circRNA biogenesis is different from the canonical splicing of linear RNAs [27]. CircRNAs are mainly generated by a process called back-splicing, where downstream exons are spliced to upstream exons in reverse order [28-31], and can be classified into three types - exonic circRNAs (ecircRNAs), retained-intron circRNAs or EIciRNAs and intronic circRNAs (ciRNAs) (Fig. 1) [11, 12, 27, 29]. In addition, circRNAs biogenesis of characteristic back-splicing reaction can be facilitated by complementary flanking Alu elements, specific splicing factors (e.g., Quaking and MBL), or exon skipping [13, 17, 27, 32-34]. Studies have also revealed that ecircRNAs, primarily localized in the cytoplasm, can mediate miRNA function, while ciRNAs, mainly found in the nucleus, can function as transcriptional regulators of their parental gene by interacting with RNA Pol II [12, 14].

CircRNAs play important roles in regulating gene expression at multiple levels via neutralization of endogenous miRNAs, protein binding, regulation of parental gene expression, or translation into functional proteins (Fig. 1) [12-14, 18, 35, 36]. As competitive endogenous RNAs (ceRNAs), circRNAs can negatively regulate miRNAs by competing for miRNA-binding sites, thereby indirectly regulating the expression of the miRNA targets [14, 37]. The most representative is a circRNA named ciRS-7 or CDR1as, which contains over 70 selectively conserved miRNA target sites, serving as miR-7 sponge [8, 14]. Likewise, cSRY that is specifically expressed in mice testes harbors 16 binding sites for miR-138 [14, 38]. In addition, circRNAs can also bind to, sequester, and transport RBPs, suggesting another process for circRNA-mediated gene regulation [39, 40]. It has been reported that circFoxo3 can suppress cell cycle by interacting with CDK2 and p21 [39]. Another study also found that circFoxo3 could regulate cardiac senescence through interacting with senescence-associated proteins (e.g., ID1 and E2F1) and stress-related proteins (e.g., HIF1a and FAK) in the cytoplasm [18]. Several studies also found that EIciRNAs and ciRNAs can regulate transcription of their parental genes [11, 12]. The circEIF3J and circPAIP2 EIciRNAs that are mainly localized in the nucleus can bind to U1 small nuclear ribonucleoproteins (snRNPs), form the EIciRNA-U1 snRNP complex, and interact with RNA Pol II in the promoter region, consequently enhancing the transcription of their parental genes [11]. On the other hand, the ci-ankrd52 ciRNA can directly interact with RNA Pol II and promote the transcription of its parental gene, *ANKRD52* [12]. Several studies also revealed that circRNAs containing internal ribosome entry site elements (IRES) or prokaryotic ribosome-binding sites can encode proteins *in vivo* or *in vitro* [30, 35, 41]. It has been found that circMbl3 could translate protein in fly heads [42]. More recently, a novel circRNA named circPINTexon2 was reported to contain short open reading frames (sORFs) that can be translated to proteins when driven by IRES in glioblastoma [43]. This discovery provides a new direction for future circRNA studies.

## 3. CircRNAs as potential biomarkers for age-related diseases

Based on their characteristics and biological functions, circRNAs have a great potential to act as essential biomarkers to predict disease progression and prognosis. The circRNAs previously identified that are potential biomarkers in age-related diseases, specifically cardio-cerebrovascular disease, neurodegenerative disease, cancer, and diabetes, are summarized in Table 1.

### 3.1 CircRNAs in cardio-cerebrovascular disease

Cardio-cerebrovascular disease (CVD) is an important age-related disease that is the most prominent cause of human mortality and morbidity worldwide. Despite the extensive amount of research for CVD, the survival of CVD patients has not significantly improved due to the lack of rapid and accurate diagnostic procedure and effective treatment. Thus, early diagnosis and proper therapeutic intervention are critical for improving the survival rates of CVD patients.

Recent studies have suggested that circRNAs may play important roles in the initiation and development of CVD [18, 44-50]. The first evidence confirming that circRNAs are involved in the regulation of heart physiology and pathology was reported by Wang *et al*. in 2016, who discovered a circRNA termed heart-related circRNA (HRCR) that can act as an endogenous miR-223 sponge and inhibit cardiac hypertrophy, decreasing the probability of heart failure [44]. Subsequently, the circMFACR/miR-652-3p/MTP18 axis was discovered to regulate cardiomyocyte apoptosis, mitochondrial fission, and myocardial infarction (MI), indicating that circMFACR may be a potential therapeutic target for cardiovascular disease [47]. Another group investigated the relationship between circRNAs and aging and found that circFoxo3, which was highly expressed in the aged hearts of both humans and mice, interacted with ID-1, E2F1, FAK, and HIF1a and retained them in the cytoplasm, limiting their anti-senescent and anti-stress roles and consequently promoting cardiac senescence [18]. On the other hand, a group from Germany and the USA focused on the role of circRNAs in atherosclerosis and found that circANRIL confers atheroprotection by controlling ribosomal RNA (rRNA) maturation and modulating the associated pathways during atherogenesis, suggesting that circRNAs can alter RNA function and ultimately influence atherosclerosis development [45]. In addition, Zhao *et al*. reported that the hsa_circ_0124644 in the peripheral blood may be a sensitive and specific biomarker for diagnosing coronary artery disease (CAD) [46], while another study presented a transcriptome-wide overview of aberrantly expressed circRNAs in CAD patients and identified hsa_circ_0001879 and hsa_circ_0004104 as novel CAD biomarkers [48].

Stroke is the leading cause of disability and death worldwide, with approximately 80% of the cases attributed to ischemia [51]. Bai *et al*. found that circDLGAP4 levels were significantly decreased in the plasma of acute ischemic stroke (AIS) patients [49]. On the other hand, Han *et al*. reported that the circHECTD1-MIR142-TIPARP axis was involved in ischemic stroke, providing translational evidence that circHECTD1 can serve as a novel biomarker and therapeutic target for stroke [52]. In addition, Zuo *et al*. found that the significantly increased levels of circFUNDC1, circPDS5B, and circCDC14A in the plasma of AIS patients were positively correlated with infarct volume, suggesting these circRNAs may be potential biomarkers for AIS diagnosis [50].

**Table 1:** CircRNAs as potential biomarkers for age-related diseases.

Diseases	Type of diseases	circRNAs	Expression	Biological function	Refs.
Cardio-cerebrovascular disease	Heart failure	circHRCR	Downregulated	Inhibits cardiac hypertrophy and heart failure by sponging miR-223	[44]
	Myocardial infarction	circMFACR	Upregulated	Mediates cardiomyocyte death via miRNA-dependent upregulation of MTP18 expression	[47]
	Cardiac senescence	circFoxo3	Upregulated	Promotes cardiac senescence by arrest ID-1, E2F1, FAK, and HIF1a in the cytoplasm	[18]
	Atherosclerosis	circANRIL	Downregulated	Controls ribosome biogenesis through binding to PES1 and modulates pathways of atherogenesis	[45]
	Coronary artery disease	hsa_circ_0124644	Upregulated	Potential diagnostic biomarker of CAD in the peripheral blood	[46]
		hsa_circ_0001879	Upregulated	A novel biomarker to diagnose CAD	[48]
		hsa_circ_0004104	Upregulated	A novel biomarker to diagnose CAD	[48]
	Stroke	circDLGAP	Downregulated	Ameliorates ischemic stroke outcomes by targeting miR-143	[49]
		circHECTD1	Upregulated	Contributes to astrocyte activation and cerebral infarction by targeting miR-142-TIPARP	[52]
		circFUNDC1	Upregulated	Biomarker for AIS diagnosis and prediction of outcomes	[50]
		circPDS5B	Upregulated	Biomarker for AIS diagnosis and prediction of outcomes	[50]
		circCDC14A	Upregulated	Biomarker for AIS diagnosis and prediction of outcomes	[50]
Neurodegenerative	Alzheimer's disease	ciRS-7	Downregulated	Regulates the expression of UBE2A by sponging miR-7	[57]
disease	Parkinson’s disease	ciRS-7	Upregulated	Modulates the α-synuclein aggregation pattern in PD by targeting miR-7	[59]
	Multiple system atrophy	circIQCK	Upregulated	Potential biomarker for MSA	[60]
		circMAP4K3	Upregulated	Potential biomarker for MSA	[60]
		circEFCAB11	Upregulated	Potential biomarker for MSA	[60]
		circDTNA	Upregulated	Potential biomarker for MSA	[60]
		circMCTP1	Upregulated	Potential biomarker for MSA	[60]
	Amyotrophic lateral sclerosis	hsa_circ_0023919	Downregulated	Blood biomarker for ALS	[62]
		hsa_circ_0063411	Upregulated	Blood biomarker for ALS	[62]
		hsa_circ_0088036	Upregulated	Blood biomarker for ALS	[62]
Cancer	Prostate cancer	circCSNK1G3	Upregulated	Promotes cell growth by interacting with miR-181	[65]
		circAMOTL1L	Downregulated	Facilitates cell migration and invasion through binding miR-193a-5p	[66]
		circ_0044516	Upregulated	Promotes prostate cancer cell proliferation and metastasis and serves as a potential biomarker	[67]
	Breast cancer	circCNOT2	Upregulated	A useful biomarker to choose the right type of therapy or to monitor breast cancer	[68]
		circEPSTI1	Upregulated	Regulates cell proliferation and apoptosis of TNBC by targeting BCL11A via miR-4753/6809	[69]
		hsa_circ_001783	Upregulated	Correlates with tumor burden and serves as a novel prognostic and therapeutic target for breast cancer	[70]
	Colorectal cancer	circKLDHC10	Upregulated	Potential circulating biomarker for CRC diagnosis	[56]
		circ_0001178	Upregulated	Promising biomarker for liver metastases from CRC	[71]
		circ_0000826	Upregulated	Promising biomarker for liver metastases from CRC	[71]
		circCCDC66	Upregulated	Predictive biomarker for CRC detection and prognosis	[72]
Diabetes	Diabetes	hsa_circ_0054633	Upregulated	Circulating diagnostic biomarker for pre-diabetes and T2DM	[77]
		hsa_circ_11783-2	Downregulated	Closely related to T2DM and might be novel therapeutic targets in diabetes	[78]
	Diabetic retinopathy	circ_0005015	Upregulated	Facilitates retinal endothelial angiogenic function via sponging miR-519d-3p	[80]
		circHIPK3	Upregulated	Regulates retinal endothelial cell function and vascular dysfunction by sponging miR-30a	[81]
	Diabetic cataract	circKMT2E	Upregulated	Involves in the pathogenesis of diabetic cataract	[82]
Other diseases	Rheumatoid arthritis	hsa_circ_0044235	Downregulated	Potential diagnostic biomarker of RA patients	[86]
	Osteoporosis	circRUNX2	Downregulated	Promotes the expression of osteogenic differentiation-related proteins by sponging miR-203	[90]
		circ_0002060	Upregulated	Potential diagnostic biomarker and therapeutic target in osteoporosis	[91]

### 3.2 CircRNAs in neurodegenerative disease

CircRNAs were also found to be highly expressed in the brain compared to other tissues, prompting several researchers to investigate their association with nervous system diseases [53, 54]. In one study, the total circRNA expression significantly increased in the aging central nervous system of *Drosophila*, suggesting that circRNAs may serve as aging biomarkers [55]. In addition, a recent study reported that exosomal circRNAs are capable of traversing the blood-brain barrier (BBB), making them perfect candidates as potential diagnostic tools for neurodegenerative disease [56].

One well-known example is the ciRS-7 (or CDR1as), a circRNA sponge and an inhibitor of miR-7 [57-59]. Dysregulated ciRS-7-miR-7 interaction was discovered in the hippocampus of Alzheimer's disease (AD) patients [57]. In addition, Lukiw *et al*. found that ciRS-7 deficiency resulted in the decreased expression of selective miR-7 targets, such as the AD-associated target, *UBE2A* [57]. Subsequently, this conjecture was confirmed by another study, implying that ciRS-7 may serve as an effective target for AD treatment [58]. Interestingly, another miR-7 target called α-synuclein is implicated in the pathophysiology of Parkinson’s disease (PD), suggesting that ciRS-7 also plays a role in modulating the α-synuclein aggregation pattern in PD [59]. Another research group performed circRNA sequencing of the brain samples from multiple system atrophy (MSA) patients and identified five circRNAs, namely IQCK, MAP4K3, EFCAB11, DTNA, and MCTP1, that were overexpressed in the white matter of the cortical tissue [60]. Amyotrophic lateral sclerosis (ALS) is a fatal neurodegenerative disease mainly characterized by muscle atrophy, speech difficulties, and respiratory insufficiency [61]. To identify biomarkers for ALS, Dolinar *et al*. compared the circRNA alterations in the leukocyte samples of ALS patients and healthy controls, and identified three circRNAs, has circ 0023919, hsa_circ_0063411, and has circ 0088036, as potential blood-based biomarkers of ALS [62].

### 3.3 CircRNAs in cancer

Age is the single most important determinant for risk of cancer [63]. Although malignant tumors can occur at all ages, cancer disproportionately strikes individuals aged 65 years and older [64]. However, the primary cause for major age-related cancers (e.g., prostate, breast, colorectal) remains unknown [63]. Therefore, it is important to emphasize the need for research focusing on the prevention of age-related cancer and the planning of treatment and care for elderly patients.

There is mounting evidence that circRNAs play critical roles in the regulation of cancer development and progression [56, 65-72]. Prostate cancer is one of the most common aggressive tumors in elderly men [73]. Recently, Chen *et al*. found that circCSNK1G3 can promote prostate cancer cell proliferation at least partially through interaction with miR-181b/d [65]. Another study revealed that circAMOTL1L was downregulated in human prostate cancer and circAMOTL1L-miR-193a-5p interaction facilitated cancer cell migration and invasion [66]. In addition, the exosomal circ_0044516 in the blood of prostate cancer patients was found to play an important role in cell survival and metastasis, suggesting its significance as a potential biomarker for prostate cancer [67].

On the other hand, breast cancer is the most frequently diagnosed cancer in elderly women [74, 75]. CircCNOT2 was proven to be a useful biomarker for choosing the right type of cancer therapy and monitoring breast cancer in a minimally invasive manner [68]. Another study reported that circEPSTI1 can function as a positive regulator of cell proliferation and apoptosis in triple-negative breast cancer (TNBC) by targeting BCL11A via miR-4753/6809 and act as an independent prognostic biomarker for survival in TNBC patients [69]. In addition, a higher level of hsa_circ_001783, which was significantly correlated with heavier tumor burden in breast cancer patients, may serve as a novel prognostic and therapeutic target for breast cancer [70].

The involvement of circRNAs in colorectal cancer (CRC), has a high incidence in both elderly men and women, has also been reported [56, 63, 71, 72]. Li *et al*. demonstrated that circKLDHC10, which was enriched and stable in exosomes, may serve as a potential circulating biomarker for CRC diagnosis [56]. In a circRNA sequence study, Xu *et al*. discovered that circ_0001178 and circ_0000826 were significantly upregulated in CRC metastatic tissues and may be used as biomarkers for liver metastases from CRC [71]. Furthermore, the findings of Hsiao *et al*. indicated that the elevated expression level of circCCDC66 was a good predictive biomarker for CRC detection and prognosis [72]. Collectively, these studies suggest that circRNAs can be potential biomarkers for cancer diagnosis and novel targets for cancer treatment.

### 3.4 CircRNAs in diabetes

Diabetes is an age-related metabolic disorder involving insulin secretion abnormalities and defects due to the action of insulin against its target tissues [76]. There were nearly 410 million diabetic patients around the world in 2015, of which approximately 46.5% have not yet been diagnosed [77]. Furthermore, the high morbidity and mortality rates in diabetic patients impose a huge social and economic burden on human society, therefore, early diagnosis and intervention are urgently needed. In a recent study, the level of hsa_circ_0054633 in the peripheral blood was associated with diabetes and may serve as a circulating diagnostic biomarker for pre-diabetes and type 2 diabetes mellitus (T2DM) [77]. Another research group verified that hsa_circ_11783-2 was closely related to T2DM, indicating that this circRNA may be a novel therapeutic target for diabetes, indicating that this circRNA might serve as a potential diagnostic biomarker of RA [78].

In the advanced stages of diabetes, patients often experience various vascular complications, which are the major causes of disability and high mortality among diabetic patients [79]. In a recent study, circ_0005015 was verified to be upregulated in the plasma, vitreous sample, and fibrovascular membranes of diabetic retinopathy (DR) patients [80]. Likewise, circHIPK3 expression was also significantly upregulated in diabetic retinas; silencing of circHIPK3 *in vivo* alleviated retinal vascular dysfunction, suggesting that this circRNA is a potential target for controlling proliferative DR [81]. Diabetic cataract (DC) is the leading cause of non-traumatic visual impairment and blindness worldwide. Fan *et al*. demonstrated that circKMT2E was involved in the pathogenesis of DC, providing a new target for the non-surgical treatment strategies [82].

### 3.5 CircRNAs in other age-related diseases

Rheumatoid arthritis (RA), which affects approximately 1% of the world’s population, is a chronic systemic autoimmune disease characterized by the debilitating in?ammation and destruction of the joints [83-85]. Early diagnosis and proper treatment can effectively relieve the pathogenetic condition in RA patients. Growing evidence has shown that dysregulated expression of circRNAs was associated with RA [86, 87]. For example, Luo *et al*. found that the hsa_circ_0044235 in the peripheral blood was significantly downregulated in RA patients, indicating that this circRNA might serve as a potential diagnostic biomarker of RA [86].

The relationship between circRNAs and osteoporosis, a multifactorial and common bone disease characterized by an increased risk of bone fracture (BF) due to the fragility and reduction of bone mass, has also been explored [88, 89]. One study reported that circRUNX2 can sponge miR-203 and promote the expression of osteogenic differentiation-related proteins, such as RUNX2, OCN, OPN, and BSP, consequently preventing osteoporosis [90]. Another study explored the expression of circRNAs using microarray analysis and identified circ_0002060 as a potential diagnostic biomarker and therapeutic target for osteoporosis [91].

## 4. Conclusion and perspectives

In recent years, circRNAs have gradually become one of the most prominent targets in the field of transcriptomics because of their critical roles in the regulation of gene expression and development of several diseases. The characteristic stability, abundance, and tissue-specific expression of circRNAs confer them great potential for use as biomarkers of various diseases [8, 16, 17]. Notably, circRNAs can exist in the exosomes and plasma due to their excellent stability, thus, providing a more convenient way for diagnosing pathologies [56, 92, 93]. Here, we presented some examples of circRNAs that are involved in age-related diseases that may serve as potential biomarkers. However, further studies specifically aimed at elucidating the function of circRNAs in the aging process are still required.

Studies investigating the diagnostic implications and significance of circRNAs are limited, since many primarily focus on the differential expression of circRNAs. Potential biomarker applications will require an in-depth knowledge of the mechanism regarding how circRNAs are changing in relation to disease development and progression. Furthermore, the reliability and sensitivity of using circRNAs as biomarkers requires thorough validation. Finally, detecting circRNAs in the blood cells or exosomes is more expensive and time-consuming than existing checks, thus, the improvement of existing clinical methods for RNA detection is urgently needed.

In conclusion, further investigations regarding the function and mechanism underlying the associations between circRNAs and age-related diseases are required. In addition, future studies may identify additional promising circRNA biomarkers for potential clinical use.
